# Diagnostic Mycology Laboratories Should Have a Central Role for the Management of Fungal Disease

**DOI:** 10.3390/jof8121285

**Published:** 2022-12-08

**Authors:** Narda Medina, Ana Alastruey-Izquierdo, Danicela Mercado, David W. Denning, Eduardo Arathoon, Juan Luis Rodriguez-Tudela

**Affiliations:** 1 Asociación de Salud Integral, Guatemala 01001, Guatemala; 2 Global Action for Fungal Infections, 1208 Geneva, Switzerland; 3 Mycology Reference Laboratory, National Centre for Microbiology, Instituto de Salud Carlos III, 28222 Madrid, Spain; 4 Centro de Investigación Biomédica en Red de Enfermedades Infecciosas (CIBERINFEC), Instituto de Salud Carlos III, 28222 Madrid, Spain; 5 Clínica Familiar “Luis Ángel García”/Hospital General San Juan de Dios, Guatemala 01001, Guatemala; 6 Manchester Fungal Infection Group, Manchester Academic Health Science Centre, The University of Manchester, Manchester M13 9PL, UK

**Keywords:** public health, Latin America, HIV, opportunistic infections, tuberculosis, histoplasmosis, cryptococcosis

## Abstract

The absence of awareness of fungal diseases as part of the differential diagnosis in at-risk populations has severe consequences. Here, we show how the active role of laboratories can improve patients’ survival. Recently, major advances have been made in non-culture-based assays for fungal diseases, improving accuracy and turnaround time. Furthermore, with the introduction of proficiency control systems, laboratories are an easily monitored environment with good analytical accuracy. Diagnostic packages for opportunistic infections can overcome many deficiencies caused by the absence of awareness. In Guatemala, to make diagnosis accessible, we set up a diagnostic laboratory hub (DLH) providing screening for cryptococcosis, histoplasmosis and tuberculosis to a network of 13 healthcare facilities attending people living with HIV (PLWHIV). In two years, we screened 2127 newly HIV-diagnosed patients. The frequency of opportunistic infections was 21%, rising to 30.3% in patients with advanced HIV disease (<200 CD4); 8.1% of these patients had more than one infection. With the implementation of this diagnostic package, mortality decreased by 7%, a key goal of many public health interventions. Screening for serious infection in high-risk populations can partially overcome training or experiential deficiencies among clinicians for life-threatening fungal diseases.

## 1. Introduction

Accurate diagnosis is a key component of medical practice. There are two errors associated with human judgment: bias and noise. Bias is a systematic error that supports a prediction. Although there are many types of bias, its main characteristic is that the knowledge needed to solve the problem is incomplete, and the final judgement is systematically oriented to the same and wrong decision. However, noise is a random error [1], which depends on the person making the decision. Both bias and noise affect clinical decision making.

Clinicians have a central role in health systems. They interact with patients, order diagnostic and monitoring tests, reach diagnoses and decide on treatments. For most conditions, laboratory tests are essential, and without them, the diagnosis remains elusive or uncertain. Without a definitive diagnosis, clinicians use empirical therapies for patients with variable success rates and frequent penalties in terms of adverse effects. In addition, empirical therapies usually combine antimicrobials to try to cover all the possible microorganisms possibly responsible for infection; the development of resistance is likely if the chosen antimicrobials are not fully active against the infecting or colonising microorganisms. Bias and noise are inherently associated with this style of empirical medical practice.

Fungal diseases have been neglected for years. Training is limited, and there is significant noise when a clinician evaluates a patient who could have a fungal infection. As fungal diseases are infrequently included in the differential diagnosis of many clinical settings, laboratory tests are not ordered, the fungal disease is not diagnosed, and often, only antibacterial therapy is given. The consequences for patients are serious, often fatal.

This situation was perhaps tolerable decades ago when only a few antifungal treatments were available and when fungal diagnostics were slow and insensitive. However, currently, although the situation is still far from being ideal, we have several rapid and inexpensive diagnostic assays and multiple antifungals approved and regarded as essential by the World Health Organization (WHO) with others in the pipeline [2]. In addition, the WHO has recently released the first *Fungal Priority Pathogen List*; in this document, the WHO prioritizes (among other actions) improving mycology diagnostic capacity to manage fungal infections and to incorporate fungal diseases and priority pathogens in medical (clinical) and public health educative programs and curricula at all levels of training [3].

The purpose of this article is to put forward initiatives to decrease the bias and the noise in fungal diseases.

## 2. The Diagnostic Mycology Laboratory and Its Role

The clinical spectrum of fungal diseases is broad, from self-limited diseases to life-threatening infections, and many medical specialties deal with them. In the 1970s, the focus on fungal diseases was on those infections affecting the skin and subcutaneous tissues, endemic diseases caused by primary pathogens and opportunistic infections in leukaemia and transplant recipients. However, when the HIV pandemic appeared, fungal diseases became a killer for those patients infected by the virus. *Pneumocystis jirovecii*, *Cryptococcus neoformans*, *Histoplasma capsulatum*, *Talaromyces marneffei*, *Candida* spp. and *Aspergillus* spp. cause over a million deaths worldwide. Furthermore, advances in medicine have increased the number of immunocompromised patients, and fungal diseases have taken the spotlight as one of the main limitations in these new effective therapies to treat cancer, avoid organ transplant rejection or provide a better outcome for other immune disorders. Thus, Mucorales, *Fusarium* spp., *Scedosporium* spp. And other multiresistant pathogens such as *Lomentospora prolificans* have emerged as lethal threats to patients. Secondary resistance to antifungals has increased in recent years, and so *Candida glabrata* and *Aspergillus fumigatus* are becoming increasingly difficult to treat. New emerging resistant fungi, such as *Candida auris,* have ‘invaded’ hospitals across the world, causing nightmare outbreaks that are difficult to control and eliminate [4,5,6]. Even some previously easy-to-treat skin infections are now difficult to manage because of the emerging *Trichophyton indotineae* showing terbinafine resistance [7,8].

Fungal pathogens have often shifted more quickly than clinical awareness in all specialties. Many deep fungal infections in immunocompromised patients are asymptomatic in the early stages, and when symptoms do appear, they are not distinctive [9]. Limited knowledge means that fungal diseases are infrequently included in the differential diagnosis (until it is too late). Diagnostics tests in at-risk populations are absolutely necessary to reach or exclude diagnoses of fungal diseases. As an example, many decision algorithms suggest thinking “fungal disease” when a patient with a fever does not respond to antibacterials, which requires waiting several (valuable) days to determine the response. For fungal diseases, early diagnosis and the institution of active antifungal therapy are directly linked to improved survival [10,11]. The FPPL launched by the WHO aims to focus and drive further research and policy interventions to strengthen the global response to fungal infections and identify major areas for action, including improving diagnostic capacity and clinical training in fungal diseases worldwide [3].

Given the need for precise diagnosis of fungal diseases, the central role should be taken by the microbiology laboratory. Nowadays, the diagnostic landscape has been shifted from direct exams and cultures to rapid testing by means of lateral flow assays (LFA), latex agglutination, enzyme immunoassays (EIA), colorimetric, real-time PCR, matrix-assisted laser desorption/ionization-time of flight (MALDI-TOF), sequencing and metagenomics as well as resistance detection to antifungals and therapeutic drug monitoring (TDM). Many commercial tests are approved for clinical use by the European Medical Agency of the European Union and the Food and Drug Administration of the USA. This has allowed performance comparison of the techniques, making it possible to identify the most reliable tools to diagnose specific diseases. The WHO’s *Essential Diagnostic List* includes most of the techniques currently employed to diagnose fungal diseases [12]; however, most of these tests are unavailable in many low- and middle-income countries (LMIC). One reason for the lack of tests is that few clinicians are incompletely trained in fungal diseases, and there are no diagnostic requests, leading to laboratories not offering those tests—a manifestation of ‘market failure’. With an adequate diagnosis, many fungal diseases are ‘invisible’, and the incidence or prevalence remains unknown. Inevitably, public health authorities ignore unseen problems, and thus, no warnings are made to governments; no budgets are allocated, and the neglected situation of fungal diseases remains neglected. Patients suffer death and morbidity, which is often treatable, and the circular problem of neglect remains unsolved.

Biases related to fungal diseases among clinicians can be solved with extensive education linked with the provision of a diagnosis. However, training takes a long time, and it is influenced by many variables, including the quality of the teaching system. There are available tools that could help, such as guidelines for fungal disease and at-risk populations [13,14,15,16,17]. However, they need adoption by countries as national guidelines.

One promising tool is the development of artificial intelligence (AI) clinical decision support systems, which could be developed for specific populations at risk, such as those with advanced HIV disease. Homogeneous risk groups with a limited number of opportunistic infections will be easier to support with AI. These decision support tools would be relatively affordable to develop, test and validate in a short time. However, extensive training in its use would be another limitation for quick implementation.

Laboratories are much more controlled environments that can be accredited according to international mores and monitored with strong quality assurance procedures. New automated laboratory systems require a lower number of workers facilitating continuous education programs and lower variability of the entire diagnostic process. Without any doubt, training on fungal diseases must be reinforced everywhere, as the WHO has stated. However, we propose a new health system that could quickly decrease the bias and the noise that fungal diseases cause.

**Figure 1:**
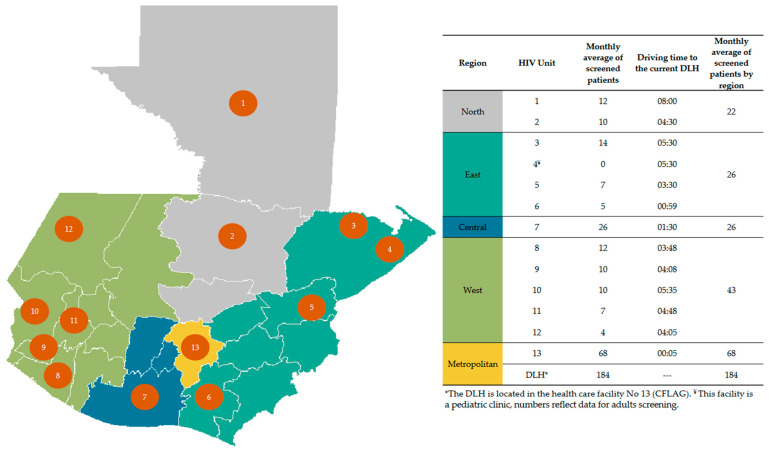
Summary of the HIV units of the network, average number of screened patients in every location, and driving time to the diagnostic laboratory hub.

The Global Action for Fungal Infections (GAFFI) established a partnership with the Asociación de Salud Integral in Guatemala in 2015. Since then, we have conducted a prospective study implementing a package of care to screen for cryptococcosis, histoplasmosis and tuberculosis in PLWHIV. The main objective of the programme was to test if improved access to diagnosis could decrease mortality in PLWHIV. To make diagnosis accessible, we set up a diagnostic laboratory hub (DLH)) at the HIV facility partner, Clínica Familiar Luis Angel García—Asociación de Salud Integral, and established a network called FUNGIRED comprising 13 of 16 HIV units in the country. Overall, the 13 HIV units covered care for ~60% of the national cohort (Figure 1). The DLH provided the diagnostic package of care for opportunistic infections to FUNGIRED. The work started in January 2017 and was completed in December 2019. Three groups of patients were included: (i) newly diagnosed HIV patients, (ii) those out of care but returning to care, and (iii) those on ARV but with symptoms of opportunistic infection disease. All patients enrolled were intended to be screened for cryptococcosis, histoplasmosis, non-tuberculous mycobacterial infection and tuberculosis, regardless of the CD4 count. The methods used and specific results have been previously described [18,19,20]. In summary, between 2017 and 2018, there were 2127 newly diagnosed HIV patients, which means that the network included 58.3% of all new cases reported in the country [20]. The rate of advanced HIV disease was 52%, but 65.8% among the Mayan population. The global rate of fungal disease was 21%. Among those with opportunistic infections, coinfections comprised 8.1%, and mortality decreased from 34% to 27% in one year [20].

Data from this prospective study demonstrated that the burden of fungal diseases in Guatemala was underestimated [21,22]. In 2012, only 32 cases of histoplasmosis were recorded by the Ministry of Health, but between 2017 and 2019, the network diagnosed an average of 160 cases per year, a 5-fold times increase. As the programme covered ~60% of the PLHIV, 250 cases of histoplasmosis per year are likely, representing a 7.8-fold increase. Estimations published for the region [23] showed an incidence 4.7% lower than that found by the screening program. Similar findings were found for cryptococcosis, where a very recent analysis of cryptococcal antigen (CrAg) data showed 9.2% (IC95% 7.9–10.7%) of patients with <100 CD4/mm^3^, which is 3.2% higher than the previous global estimation [24]. The centralization of the diagnostic resources and their use for laboratory surveillance in Guatemala has enabled good estimates of the incidence of the diseases with appropriate denominators of the population at risk. These data are both comprehensible and comparable with data globally. In addition, and for the first time, we have provided data about the rate of opportunistic fungal infections for the whole population of PLWHIV looking for care and not only for those with advanced disease. This has opened up a discussion about the optimal CD4 threshold for patient screening—<200 or 350 CD4/mm^3^ [20,21].

It is important to highlight that the DLH is not a reference laboratory [18]. Reference laboratories have other functions, and diagnosis activity is often limited to the confirmation of difficult cases with tools not available everywhere because of complexity or expertise. In the future, DLHs could be co-located with reference laboratories but with the important proviso that DLHs have direct patient care as their central role so that results are communicated to clinicians at maximum speed with assured quality control. For life-threatening fungal diseases, there is a clear relationship between time to diagnosis and the survival of the patient [10,11]. Therefore, DLHs must provide quick and high-quality diagnostic services to a healthcare network providing care to a given population. In an environment where resources are limited and healthcare professionals trained in fungal disease management are the exception, screening and centralization of services are one option. However, partial decentralization may be possible for some quick and easy assays, considering that diagnosis should be as close to the patient as possible. In many settings, the incidence and prevalence of fungal diseases are relatively low, and a cost-effective approach that assures the quality of a local laboratory service may tilt toward centralisation.

In Guatemala, the DLH put in place informatic tools to enroll and track patients’ progress, receive diagnostic orders, and disseminate results. Advance notice of the number of clinical samples that the DLH will receive each day helps to organize workload, optimizing turnaround time and cost-effectiveness. Sending the results electronically to the clinician in charge of the patients made it possible to quickly establish the right treatment, which is key for a good outcome, especially if it is a life-threatening fungal disease. We also analysed the workload of the HIV units in Guatemala. Figure 1 shows the geographic distribution of the HIV units and the monthly average of patients screened by every unit (in the range of 5 to 26). This can be compared with the DLH workload (average 184). In this instance, centralization of diagnosis (with a rapid transport system) allowed for acceptable quality of the results and a cost-effective and timely approach. However, the turnaround time from sample collection to delivery of results should be optimized and audited. This was one of the drawbacks of the project performed in Guatemala, where, in some cases, the time to deliver the samples was not optimal. Considering this, we have recommended that DLHs should be located at a maximum distance of one or two hours by ground transport. Furthermore, a working scheme of 24 h/7 days for certain diagnostic tests should be adopted to decrease the turnaround time.

Because of the difficulties regarding the quick transport of clinical samples in many countries, this approach might not be ideal. In high-income countries, there is a general trend of service centralization because the well-designed transport of clinical samples is much more cost-effective. The term “bedside diagnostic tool” must be revisited because such a diagnostic device should be used without any equipment. The best examples are the HIV or COVID-19 point-of-care-test (POCTs). Without a doubt, POCTs are essential, especially for stigmatizing diseases such as HIV or sexually transmitted conditions. However, sooner or later, all results require confirmation to decide on clinical management if positive.

In the case of immediately life-threatening diseases, decentralization of diagnostics should be carefully considered. If a patient at risk (e.g., an advanced HIV disease patient) has a positive test for a life-threatening fungal disease at a primary care center, that patient needs care at a facility able to confirm the diagnosis and offer optimal treatment. POCT diagnosis in primary care could be useful from a screening point of view, especially in places where many PLHIV arrive with advanced HIV for the first time. AI apps designed for mobile phones able to read and interpret LFA devices could make primary care diagnostic services a reality by recording and linking results to patients’ other metadata in the cloud [25,26].

Centralization vs. decentralization of diagnosis needs to be carefully designed, seeking a combination approach, probably with a network of laboratories. LFA techniques for the diagnosis of life-threatening fungal diseases can be located at different healthcare facilities, from primary to reference care, depending on the incidence of the disease, but always considering that, ideally, patients should be treated locally. Confirmatory tests, if they are needed, should be located at a DLH where trained people, quality control and proficiency systems warrant the reliability and cost-effectiveness of the system.

Screening is unrealistic for non-life-threatening infections. However, for candidemia or disseminated candidosis, aspergillosis, cryptococcosis, histoplasmosis, *Pneumocystis jirovecii* pneumonia, talaromycosis, etc., screening with tests with high sensitivity and specificity and a quick turnaround time offers great advantages. In addition, variability in clinical practice—noise—makes this desirable. The higher frequency of certain fungal diseases in specific underlying diseases makes such implementation of screening strategies or packages of care easier.

Our screening experience with PLWHIV in Guatemala found a remarkably high incidence of opportunistic infections (21%) [20]. Differentiating tuberculosis and histoplasmosis was extremely difficult on clinical grounds, and some patients had both conditions. Among those with AHD, the incidence of opportunistic infections was 30.3%, and 10.1% had dual or triple infections. It seems wise to provide a package of diagnosis, considering the immunological situation of the patient irrespective of clinical suspicion, for this patient group.

An example of the noise from clinicians is shown in Table 1, which shows the increasing number of patients enrolled in the first two years of the program. As clinicians became aware that opportunistic infections were being found in patients that they did not suspect of having a particular infection, the number of enrolled patients and samples grew. Clinical evaluation was supported with rapid access to diagnosis. Implementing a package of care in Guatemala reduced the noise and some diagnostic biases, enabling the right treatment to be given and decreasing the mortality in the cohort [20]. It is probable that implementation in other settings would obtain similar results in a short time frame [20].

Screening approaches have been successfully explored for other underlying diseases, including aspergillosis, in hematological patients not taking mold-active antifungal prophylaxis [27,28,29]. Screening in intensive care with beta D glucan reduced unnecessary empiricism [30]. In areas of high endemicity for coccidioidomycosis, screening for this disorder in newly admitted patients with pneumonia would enable antifungal therapy to be given and intravenous antibiotics to be stopped [31].

Access to diagnosis has at least a dual use: the potential to save patients’ lives and indirect training for clinicians. No doubt that such an unexpected result for clinicians is a powerful educational tool that improves care for other patients as well. It also offers the opportunity to tailor antimicrobial use contributing to antimicrobial resistance control [32].

In summary, in settings where there is limited clinical expertise in fungal diseases, we recommend setting up decentralised packages of care based on diagnostic laboratories. Commercial kits including LFAs, EIAs and PCRs are easy systems to put in place and cost-effective with a minimum number of samples per week. Other techniques, including identification by MALDI-TOF, sequencing, and metagenomics, require highly trained staff and sophisticated and costly equipment, and thus, centralized services are much more appropriate. Finally, access to fungal disease diagnosis by means of a network of decentralized DLHs and specific screening programs for some underlying diseases would quickly decrease the bias and the noise due to the lack of training and judgement variability that is so common in the management of life-threatening fungal infections. In addition, DLHs would quickly establish the local burden of the disease allowing appropriate resource allocation for both diagnostic and treatment services—from primary care to reference centres.

## Figures and Tables

**Table 1:** Numbers of patients enrolled in the OI program between 2017 and 2018.

Category	2017	2018
Patients screened	1953	2292
With OI	317 (16.2%)	399 (17.4%)
Frequency per OI		
Histoplasmosis	99 (31.2%)	128 (32.1%)
Cryptococcosis	59 (18.6%)	79 (19.8%)
Tuberculosis	114 (36%)	135 (33.8%)
Coinfections	31 (9.8%)	31 (7.8%)
Total	317 (100%)	399 (100%)

## Data Availability

Data presented in this study are available on request from the corresponding author.
